# Supplementary material for: Development of the Hypertension Index Model in General Adult Using the Korea National Health and Nutritional Examination Survey and the Korean Genome and Epidemiology Study
Source: J Pers Med. 2021 Sep 28;11(10):968. doi: 10.3390/jpm11100968 (PMC8540826; doi:10.3390/jpm11100968)
Supplement: Supplementary file 1 [file jpm-11-00968-s001.zip › jpm-1326785-supplementary.pdf]

Supplementary Materials

Development of the Hypertension Index Model in General Adult Using the Korea National Health and Nutritional Examination Survey and the Korean Genome and Epidemiology Study

Myung Jae Seo [1], Sung Gyun Ahn [2], Yong-Jae Lee [3] and Jong Koo Kim [1,*]

1 Department of Family Medicine, Yonsei University Wonju College of Medicine, Wonju 26426, Republic of Korea; smjae@yonsei.ac.kr (M.J.S.)
2 Division of Cardiology, Department of Internal Medicine, Yonsei University Wonju College of Medicine, Wonju 26426, Republic of Korea; sgahn@yonsei.ac.kr
3 Department of Family Medicine, Yonsei University College of Medicine, Seoul 06273, Republic of Korea; ukyjhome@yuhs.ac
* Correspondence: kimjk214@yonsei.ac.kr; Tel.: +82-33-741-1416

Table S1. References in the literature-based search for variables.

Variables	Journal	Author	Year of publication
Age, *years*	Hypertension and aging [1]	Buford TW	2016
Sex, *n*	Gender Differences in Epidemiology, Pathophysiology, and Treatment of Hypertension [2]<br>Prevalence of hypertension in China [3]	Di Giosia P.<br>Gao Y	2018<br>2013
Ethnicity, *n*	Trends in prevalence, awareness, treatment, and control of hypertension in the United States [4]	Hajjar I	2003
Education status, *n*<br>Elementary school<br>Middle school<br>High school<br>University	Education, literacy, and health: Mediating effects on hypertension knowledge and control [5]<br>Socioeconomic status and hypertension [6]	Pandit A.U.<br>Leng B	2009<br>2015
Income status, *n*<br>1st quartile<br>2nd quartile<br>3rd quartile<br>4th quartile	Socioeconomic status and hypertension [6]	Leng B	2015
Diabetes mellitus, *n*	Hypertension and diabetes mellitus : coprediction and time trajectories [7]	Tsimihodimos V	2018
Dyslipidemia, *n*	Dyslipidemia and the Risk of Developing Hypertension in a Working-Age Male Population [8]	Otsuka T	2016
Cancer, *n*	Hypertension and breast cancer risk [9]<br>Hypertension and risk of prostate cancer [10]<br>Blood pressure and kidney cancer risk [11]	Han H<br>Liang Z<br>Hidayat K	2017<br>2016<br>2017
Alzheimer disease, *n*	Association between blood pressure and Alzheimer disease [12]	Gabin, J.M.	2017
Parkinson disease, *n*	Association of blood pressure and hypertension with the risk of Parkinson disease [13]	Qiu C	2011
Non-alcoholic fatty liver disease, *n*	Hypertension and Nonalcoholic Fatty Liver Disease Proven by Transient Elastography [14]	Wang Y	2016
Smoking, *pack-years*	Association between smoking and blood pressure [15]	Primatesta P	2001
Alcohol consumption, *g/week*	Effects of alcohol reduction on blood pressure [16]<br>Alcohol consumption and risk for hypertension in middle-aged Japanese men [17]	Xin X<br>Nakanishi N.	2001<br>2001
Exercise, *n*	Progressive resistance exercise and resting blood pressure [18]	Kelley GA	2000

	Effect of aerobic exercise on blood pressure [19]	Whelton SP	2002
Diet<br>Total energy intake, *kcal*<br>Sodium intake, *mg*<br>Potassium intake, *mg*	Effects on blood pressure of reduced dietary sodium and the Dietary Approaches to Stop Hypertension (DASH) diet [20]<br>Effects of diet and sodium intake on blood pressure [21]<br>Dietary approaches to prevent and treat hypertension [22]	Sacks FM<br>Vollmer WM<br>Appel L.J.	2001<br>2001<br>2006
Sleep duration, *hours*	Short sleep duration is associated with hypertension risk among adults [23]	Wang Q	2012
Menopause, *n*	The influence of menopause on blood pressure [24]<br>Menopause-related blood pressure increase and its relationship to age and body mass index [25]	Staessen J<br>Zanchetti A	1989<br>2005
Oral contraceptive, *n*	Prospective study of oral contraceptives and hypertension among women in the United States [26]	Chasan-Taber L.	1996
Body mass index, *kg/m2*	Influence of weight reduction on blood pressure [27]<br>Long-term effects of weight loss and dietary sodium reduction on incidence of hypertension [28]	Neter JE<br>He J	2003<br>2000
Waist circumference, *cm*	The relationship of waist circumference to blood pressure [29]	Siani A	2002
Systolic blood pressure, *mmHg*	Assessment of frequency of progression to hypertension in non-hypertensive participants in the Framingham Heart Study [30]	Vasan RS	2001
Diastolic blood pressure, *mmHg*	Assessment of frequency of progression to hypertension in non-hypertensive participants in the Framingham Heart Study [30]<br>High-normal blood pressure progression to hypertension in the Framingham Heart Study [31]	Vasan RS<br>Leitschuh M	2001<br>1991
White blood cell, *thousand/μL*	Relationship between white blood cell count and incident hypertension [32]	Shankar A	2004
Hemoglobin, *g/dL*	Association of hematocrit with blood pressure and hypertension [33]	EmamianM	2017
Fasting plasma glucose, *mg/dL*	The association between fasting plasma glucose and glycated hemoglobin in the prediabetes range and future development of hypertension [34]	Geva M	2019
Total cholesterol, *mg/dL*	A prospective study of plasma lipid levels and hypertension in women [35]	Sesso HD	2005

Triglyceride, *mg/dL*	Triglycerides and triglycerides to high-density lipoprotein cholesterol ratio are strong predictors of incident hypertension [36]	Tohidi M.	2012
Creatinine, *mg/dL*	A prospective study of blood pressure and serum creatinine [37]	Perneger TV	1993
Liver enzyme, *IU/L*<br>Aspartate aminotransferase<br>Alanine aminotransferase<br>Gammaglutamyl trasferase	Gamma-glutamyltransferase is a predictor of incident diabetes and hypertension [38]	Lee DH	2003
C-reactive protein, *mg/dL*	C-reactive protein and the risk of developing hypertension [39]<br>Multiple biomarkers and the risk of incident hypertension [40]	Sesso HD<br>Wang TJ	2003<br>2007
Interleukin-6, *ng/mL*	Inflammation and hypertension: the interplay of interleukin-6, dietary sodium and the renin-angiotensin system in humans [41]	Chamarthi B	2011
Aldosterone, *ng/dL*	Serum aldosterone and the incidence of hypertension in nonhypertensive persons [42]	Vasan RS	2004
Follicle-stimulating hormone, *IU/L*	Follicle-stimulating hormone, its association with cardiometabolic risk factors [43]	Wang N	2017
Estrogen, *pg/mL*	Serum estrogen metabolites and systolic blood pressure [44]	Masi CM	2009
Testosterone, *ng/dL*	Serum Testosterone Levels and Arterial Blood Pressure [45]	Fogari R	2005
Forced vital capacity, *L*	Rate of decline of forced vital capacity predicts future arterial hypertension [46]	Jacobs D.R	2012
Forced expiratory volume during 1 s, *L*	Blood pressure increase is inversely related to lung function [47]	Engstrom G	2001

Table S2. General characteristics of internal validation dataset (KNHANES 2016–18).

	Internal validation dataset (KNHANES 2016–18) n = 10,333					
	Men			Women		
	Non-hypertension	Hypertension	*P*-value	Non-hypertension	Hypertension	*P*-value
	n = 2,245	n = 2,075		n = 3,576	n = 2,437	
Age, years	57.4 ± 0.23	63.5 ± 0.24	<.001	55.1 ± 0.17	66.1 ± 0.21	<.001
Income, n			.167			.004
1st quartile	520 (23.2)	541 (26.1)		810 (22.7)	620 (25.4)	
2nd quartile	568 (25.3)	496 (23.9)		901 (25.2)	619 (25.4)	
3rd quartile	570 (25.4)	514 (24.8)		885 (24.7)	624 (25.6)	
4th quartile	587 (26.1)	524 (25.3)		980 (27.4)	574 (23.6)	
Education, n			<.001			<.001
Elementary school	376 (16.7)	539 (26)		731 (20.4)	1343 (55.1)	
Middle school	261 (11.6)	346 (16.7)		448 (12.5)	335 (13.7)	
High school	698 (31.1)	626 (30.2)		1269 (35.5)	506 (20.8)	
University	910 (40.5)	564 (27.2)		1128 (31.5)	253 (10.4)	
Diabetes mellitus, n	317 (14.1)	591 (28.5)	<.001	260 (7.3)	626 (25.7)	<.001
Dyslipidemia, n	161 (7.2)	498 (24)	<.001	386 (10.8)	812 (33.3)	<.001
Cancer, n	83 (3.7)	92 (4.4)	.25	211 (5.9)	170 (7)	.104
Smoking, pack-years	17.9 ± 0.39	21.4 ± 0.47	<.001	0.6 ± 0.06	0.9 ± 0.1	.045
Alcohol consumption, g/week	95.3 ± 3.15	129.7 ± 3.87	<.001	19.9 ± 0.96	18.2 ± 1.31	.291
Total energy intake, kcal	2254.4 ± 18.59	2118.3 ± 19.14	<.001	1651.5 ± 10.93	1497.1 ± 12.65	<.001
BMI, kg/m2	23.8 ± 0.06	24.9 ± 0.07	<.001	23.3 ± 0.05	25.1 ± 0.07	<.001
Waist circumference, cm	85.1 ± 0.17	88.8 ± 0.19	<.001	78.5 ± 0.14	84.5 ± 0.19	<.001
Systolic BP, mmHg	116 ± 0.23	131.1 ± 0.36	<.001	112.8 ± 0.2	134.7 ± 0.36	<.001
Diastolic BP, mmHg	75.5 ± 0.17	79.5 ± 0.28	<.001	72.7 ± 0.13	77.9 ± 0.23	<.001
FPG, mg/dL	104.5 ± 0.55	112.6 ± 0.67	<.001	97.5 ± 0.31	108.3 ± 0.59	<.001
Creatinine, mg/dL	0.9 ± 0.0031	1 ± 0.0098	<.001	0.69 ± 0.0033	0.71 ± 0.0061	<.001
Total cholesterol, mg/dL	194.3 ± 0.79	182.5 ± 0.87	<.001	201.6 ± 0.63	190.9 ± 0.82	<.001
Triglyceride, mg/dL	157.1 ±	167.8 ±	.008	115.6 ±	138.9 ±	<.001

	2.72	2.95		1.32	2.02	
WBC, thousand/μL	6.3 ± 0.04	6.7 ± 0.04	<.001	5.7 ± 0.03	6.1 ± 0.03	<.001
Hb, g/dL	15.1 ± 0.03	14.9 ± 0.03	<.001	13.1 ± 0.02	13.2 ± 0.02	.017

Continuous variables are presented as mean ± standard error, and categorical variables are presented as numbers (percentage, %).
Abbreviations: KNHANES, Korea National Health and Nutrition Examination Survey; BMI, body mass index; BP, blood pressure; FPG, fasting plasma glucose; WBC, white blood cells; Hb, hemoglobin.

Table S3. Baseline characteristics of external validation dataset (KoGES).

	External validation dataset (KoGES) n =4,633					
	Men			Women		
	Non-hypertension	New-onset hypertension	*P*-value	Non-hypertension	New-onset hypertension	*P*-value
	n = 1,316	n = 847		n = 1,615	n = 855	
Age, years	49.4 ± 0.22	51.4 ± 0.3	<.001	48.4 ± 0.19	53.5 ± 0.3	<.001
Income, n			<.001			<.001
1st quartile	127 (9.7)	106 (12.5)		199 (12.3)	224 (26.2)	
2nd quartile	304 (23.1)	256 (30.2)		477 (29.5)	298 (34.9)	
3rd quartile	540 (41)	289 (34.1)		617 (38.2)	227 (26.5)	
4th quartile	345 (26.2)	196 (23.1)		322 (19.9)	106 (12.4)	
Education, n			<.001			<.001
Elementary school	185 (14.1)	173 (20.4)		452 (28)	411 (48.1)	
Middle school	233 (17.7)	203 (24)		413 (25.6)	204 (23.9)	
High school	544 (41.3)	309 (36.5)		595 (36.8)	196 (22.9)	
University	354 (26.9)	162 (19.1)		155 (9.6)	44 (5.1)	
Diabetes mellitus, n	81 (6.2)	59 (7)	.51	48 (3)	26 (3)	.999
Dyslipidemia, n	39 (3)	20 (2.4)	.481	27 (1.7)	11 (1.3)	.57
Cancer, n	4 (0.3)	0 (0)	-	40 (2.5)	21 (2.5)	.999
Smoking, pack-years	12.2 ± 0.45	13.3 ± 0.57	.156	0.3 ± 0.06	0.3 ± 0.07	.996
Alcohol consumption, g/week	113.5 ± 5.01	132.1 ± 6.48	.024	9.6 ± 0.84	7.5 ± 1.07	.134
Total energy intake, kcal	1990.3 ± 15.21	2040.4 ± 24.24	.08	1909.5 ± 17.28	1929.1 ± 26.86	.539
BMI, kg/m2	23.7 ± 0.08	24.3 ± 0.1	<.001	24 ± 0.07	25.1 ± 0.11	<.001
Waist circumference, cm	81.4 ± 0.2	83.7 ± 0.25	<.001	77.7 ± 0.22	82.6 ± 0.32	<.001
Systolic BP, mmHg	111 ± 0.29	117.9 ± 0.34	<.001	107.1 ± 0.28	118.5 ± 0.36	<.001
Diastolic BP, mmHg	74.7 ± 0.2	78.6 ± 0.23	<.001	70.9 ± 0.2	77.3 ± 0.23	<.001
FPG, mg/dL	89 ± 0.59	89 ± 0.71	.987	82.7 ± 0.43	83.8 ± 0.56	.139
Creatinine, mg/dL	1 ± 0.004	0.9 ± 0.006	.261	0.7 ± 0.003	0.7 ± 0.004	.415
Total cholesterol, mg/dL	192.1 ± 0.94	190.4 ± 1.22	.262	186.2 ± 0.84	189.5 ± 1.16	.021

Triglyceride, mg/dL	160 ± 2.72	176.5 ± 3.74	<.001	127.7 ± 1.66	143.4 ± 2.61	<.001
WBC, thousand/μL	6.7 ± 0.05	6.8 ± 0.06	.176	6.1 ± 0.04	6.3 ± 0.06	.124
Hb, g/dL	14.7 ± 0.03	14.7 ± 0.04	.197	12.4 ± 0.03	12.5 ± 0.04	.002

Continuous variables are presented as mean ± standard error, and categorical variables are presented as numbers (percentage, %).

Abbreviations: KoGES, Korean Genome and Epidemiology Study; BMI, body mass index; BP, blood pressure; FPG, fasting plasma glucose; WBC, white blood cells; Hb, hemoglobin

Table S4. Baseline characteristics of external validation dataset (KoGES) by quintiles of hypertension probabilities in Korean men.

	Quantiles of hypertension probabilities					
	Q1<br>n = 433	Q2<br>n = 432	Q3<br>n = 433	Q4<br>n = 432	Q5<br>n = 433	*P* for Trend
Range of probabilities	0.0006 – 0.0347	0.0347 – 0.0882	0.0882 – 0.1904	0.1907 – 0.3619	0.3625 – 0.9611	
Age, years	46.2 ± 0.3	47.2 ± 0.31	49.7 ± 0.38	52.2 ± 0.4	55.8 ± 0.42	<.001
Income, n						<.001
1st quartile	18 (4.2)	28 (6.5)	47 (10.9)	52 (12)	88 (20.3)	
2nd quartile	70 (16.2)	112 (25.9)	106 (24.5)	123 (28.5)	149 (34.4)	
3rd quartile	223 (51.5)	159 (36.8)	172 (39.7)	158 (36.6)	117 (27)	
4th quartile	122 (28.2)	133 (30.8)	108 (24.9)	99 (22.9)	79 (18.2)	
Education, n						<.001
Elementary school	38 (8.8)	46 (10.6)	65 (15)	77 (17.8)	132 (30.5)	
Middle school	67 (15.5)	85 (19.7)	87 (20.1)	96 (22.2)	101 (23.3)	
High school	192 (44.3)	176 (40.7)	186 (43)	168 (38.9)	131 (30.3)	
University	136 (31.4)	125 (28.9)	95 (21.9)	91 (21.1)	69 (15.9)	
Diabetes mellitus, n	18 (4.2)	23 (5.3)	30 (6.9)	23 (5.3)	46 (10.6)	.001
Dyslipidemia, n	4 (0.9)	6 (1.4)	8 (1.8)	16 (3.7)	25 (5.8)	<.001
Cancer, n	1 (0.2)	432 (100)	2 (0.5)	1 (0.2)	0 (0)	-
Smoking, pack-years	12.9 ± 0.69	12.4 ± 0.72	12.3 ± 0.81	12.8 ± 0.87	12.8 ± 0.85	.826
Alcohol consumption, g/week	86.4 ± 7.16	103.6 ± 8.1	129.2 ± 9.82	140.6 ± 10.1	144.1 ± 8.57	<.001
Total energy intake, kcal	2028.3 ± 27.88	2053.2 ± 28.44	1981.2 ± 28.66	2014.3 ± 33.36	1973 ± 29.58	.114
BMI, kg/m2	22.9 ± 0.13	23.8 ± 0.13	23.8 ± 0.13	24.3 ± 0.13	24.7 ± 0.15	<.001
Waist circumference, cm	79 ± 0.32	81.2 ± 0.33	82 ± 0.34	83.8 ± 0.33	85.3 ± 0.37	<.001
Systolic BP, mmHg	100 ± 0.34	109.4 ± 0.26	113.9 ± 0.28	118.7 ± 0.28	126.5 ± 0.3	<.001
Diastolic BP, mmHg	67.9 ± 0.3	73.9 ± 0.25	77.5 ± 0.26	79.8 ± 0.25	82.3 ± 0.23	<.001
FPG, mg/dL	86.2 ± 1.03	88.1 ± 0.88	90.2 ± 1.11	89 ± 0.94	91.4 ± 1.09	.001
Creatinine, mg/dL	0.94365 ± 0.00731	0.9338 ± 0.00784	0.95704 ± 0.00758	0.96343 ± 0.0081	0.96628 ± 0.00848	.005
Total cholesterol, mg/dL	193.9 ± 1.64	192.7 ± 1.67	192.2 ± 1.69	192.5 ± 1.6	185.8 ± 1.72	<.001
Triglyceride, mg/dL	153.2 ± 5.22	153.3 ± 4.72	159.3 ± 4.41	178 ± 5.1	188.7 ± 5.06	<.001
WBC, thousand/μL	6.6 ± 0.09	6.6 ± 0.09	6.7 ± 0.09	6.8 ± 0.09	7 ± 0.09	<.001
Hb, g/dL	14.8 ± 0.05	14.7 ± 0.05	14.7 ± 0.05	14.7 ± 0.05	14.7 ± 0.05	.14

Continuous variables are presented as median ± standard error, and categorical variables are presented as numbers (percentage, %).

Abbreviations: KoGES, Korean Genome and Epidemiology Study; Q, quintile; BMI, body mass index; BP, blood pressure; FPG, fasting plasma glucose; WBC, white blood cells; Hb, hemoglobin.

Table S5. Baseline characteristics of external validation dataset (KoGES) by quintiles of hypertension probabilities in Korean women.

	Quantiles of hypertension probabilities					
	Q1<br>n = 494	Q2<br>n = 494	Q3<br>n = 494	Q4<br>n = 494	Q5<br>n = 494	*P* for Trend
Range of probabilities	0.0007 – 0.0218	0.0219 – 0.056	0.0561 – 0.1311	0.1314 – 0.2907	0.2908 – 0.9305	
Age, years	44.4 ± 0.2	46.7 ± 0.29	48.9 ± 0.33	52.1 ± 0.35	58.5 ± 0.34	<.001
Income, n						<.001
1st quartile	21 (4.3)	46 (9.3)	74 (15)	99 (20)	183 (37)	
2nd quartile	103 (20.9)	135 (27.3)	169 (34.2)	192 (38.9)	176 (35.6)	
3rd quartile	230 (46.6)	201 (40.7)	168 (34)	138 (27.9)	107 (21.7)	
4th quartile	140 (28.3)	112 (22.7)	83 (16.8)	65 (13.2)	28 (5.7)	
Education, n						<.001
Elementary school	52 (10.5)	104 (21.1)	157 (31.8)	216 (43.7)	334 (67.6)	
Middle school	121 (24.5)	140 (28.3)	147 (29.8)	123 (24.9)	86 (17.4)	
High school	241 (48.8)	199 (40.3)	160 (32.4)	128 (25.9)	63 (12.8)	
University	80 (16.2)	51 (10.3)	30 (6.1)	27 (5.5)	11 (2.2)	
Diabetes mellitus, n	3 (0.6)	8 (1.6)	18 (3.6)	15 (3)	30 (6.1)	<.001
Dyslipidemia, n	4 (0.8)	6 (1.2)	7 (1.4)	10 (2)	11 (2.2)	.35
Cancer, n	15 (3)	14 (2.8)	15 (3)	13 (2.6)	4 (0.8)	.121
Smoking, pack-years	0.2 ± 0.06	0.3 ± 0.1	0.4 ± 0.15	0.1 ± 0.07	0.3 ± 0.1	.909
Alcohol consumption, g/week	7.9 ± 1.06	11 ± 1.65	9.3 ± 1.42	11.5 ± 2.06	4.7 ± 0.92	.013
Total energy intake, kcal	1934.4 ± 31.47	1936.7 ± 34.07	1935.9 ± 34.95	1928.3 ± 32.36	1846.1 ± 30.43	.021
BMI, kg/m2	22.8 ± 0.11	23.8 ± 0.12	24.6 ± 0.13	25.2 ± 0.15	25.5 ± 0.15	<.001
Waist circumference, cm	73.6 ± 0.32	76.9 ± 0.36	79.9 ± 0.37	82.1 ± 0.4	84.6 ± 0.41	<.001
Systolic BP, mmHg	95.4 ± 0.29	105.1 ± 0.24	111.2 ± 0.26	117.3 ± 0.26	126.4 ± 0.29	<.001
Diastolic BP, mmHg	63.2 ± 0.28	70.4 ± 0.24	74.2 ± 0.24	77.6 ± 0.25	80.3 ± 0.25	<.001
FPG, mg/dL	79.6 ± 0.5	81.3 ± 0.65	83.6 ± 0.8	84.2 ± 0.84	86.8 ± 0.9	<.001
Creatinine, mg/dL	0.71842 ± 0.00393	0.73016 ± 0.00447	0.73239 ± 0.00454	0.74332 ± 0.00483	0.76012 ± 0.0053	<.001
Total cholesterol, mg/dL	180.6 ± 1.37	182.7 ± 1.43	187.3 ± 1.51	193 ± 1.58	192.9 ± 1.65	<.001
Triglyceride, mg/dL	111.2 ± 2.13	118.9 ± 2.46	137.1 ± 3.55	141.2 ± 3.28	157.2 ± 3.72	<.001
WBC, thousand/μL	6 ± 0.07	6.1 ± 0.07	6.3 ± 0.08	6.1 ± 0.07	6.4 ± 0.08	<.001
Hb, g/dL	12.2 ± 0.05	12.3 ± 0.05	12.4 ± 0.05	12.5 ± 0.05	12.7 ± 0.05	<.001

Continuous variables are presented as median ± standard error, and categorical variables are presented as numbers (percentage, %).

Abbreviations: KoGES, Korean Genome and Epidemiology Study; Q, quintile; BMI, body mass index; BP, blood pressure; FPG, fasting plasma glucose; WBC, white blood cells; Hb, hemoglobin.

Supplementary Figure S1. Predictors for Korean men selected by NRI method.

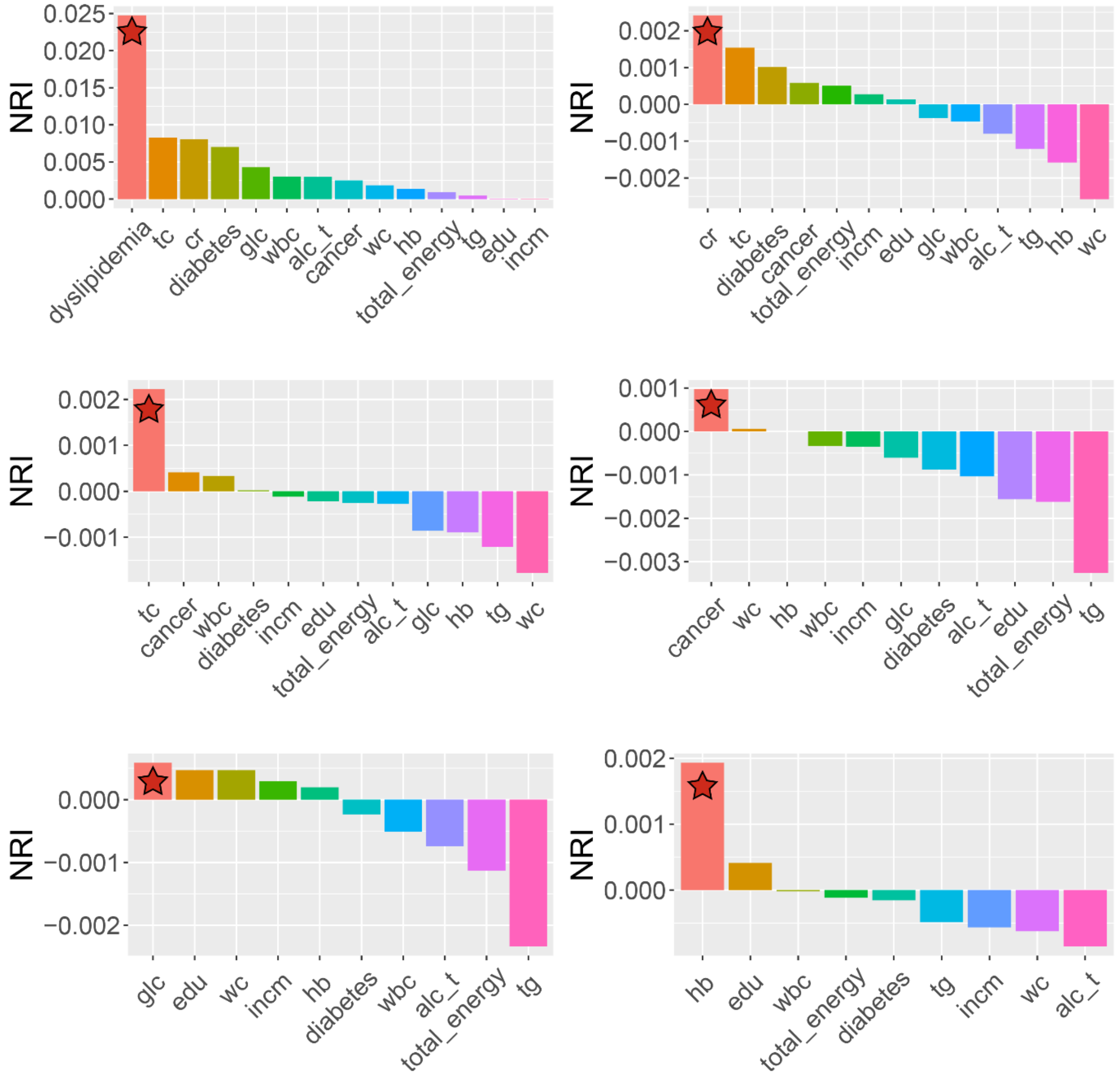

Supplementary Figure S1. Predictors for Korean men selected by NRI method (Continued).

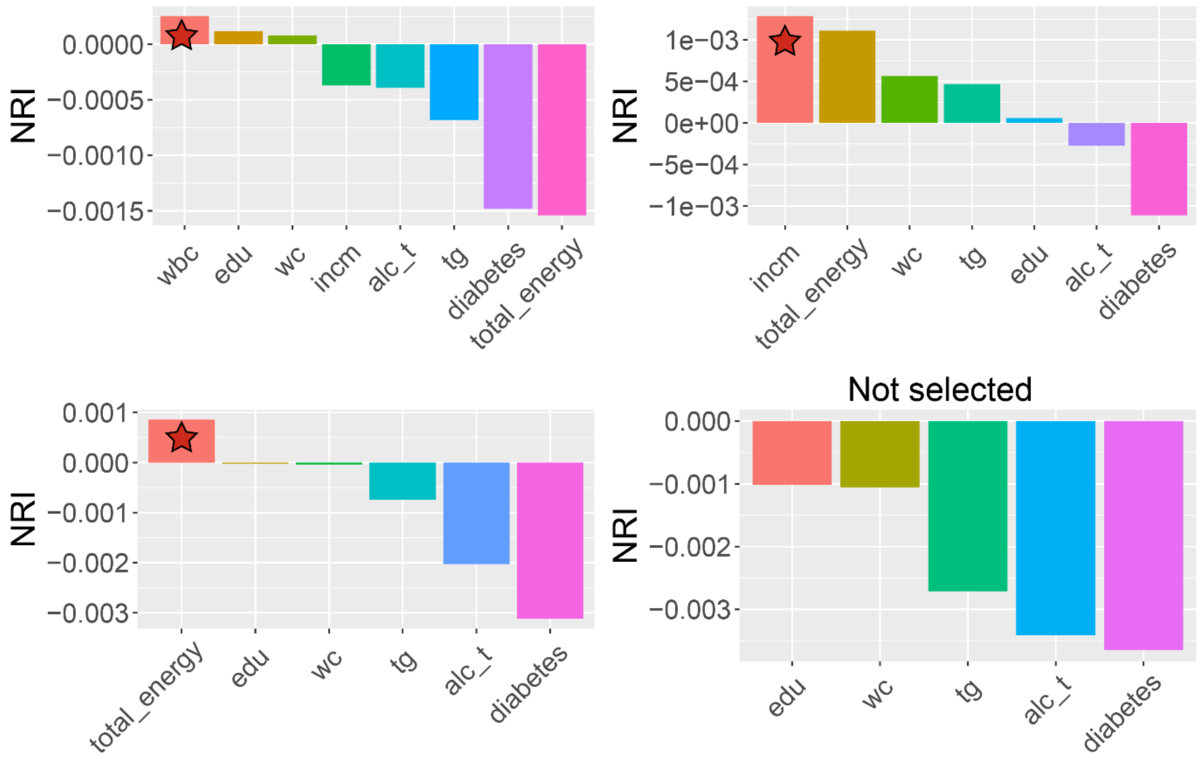

Supplementary Figure S2. Predictors for Korean women selected by NRI method.

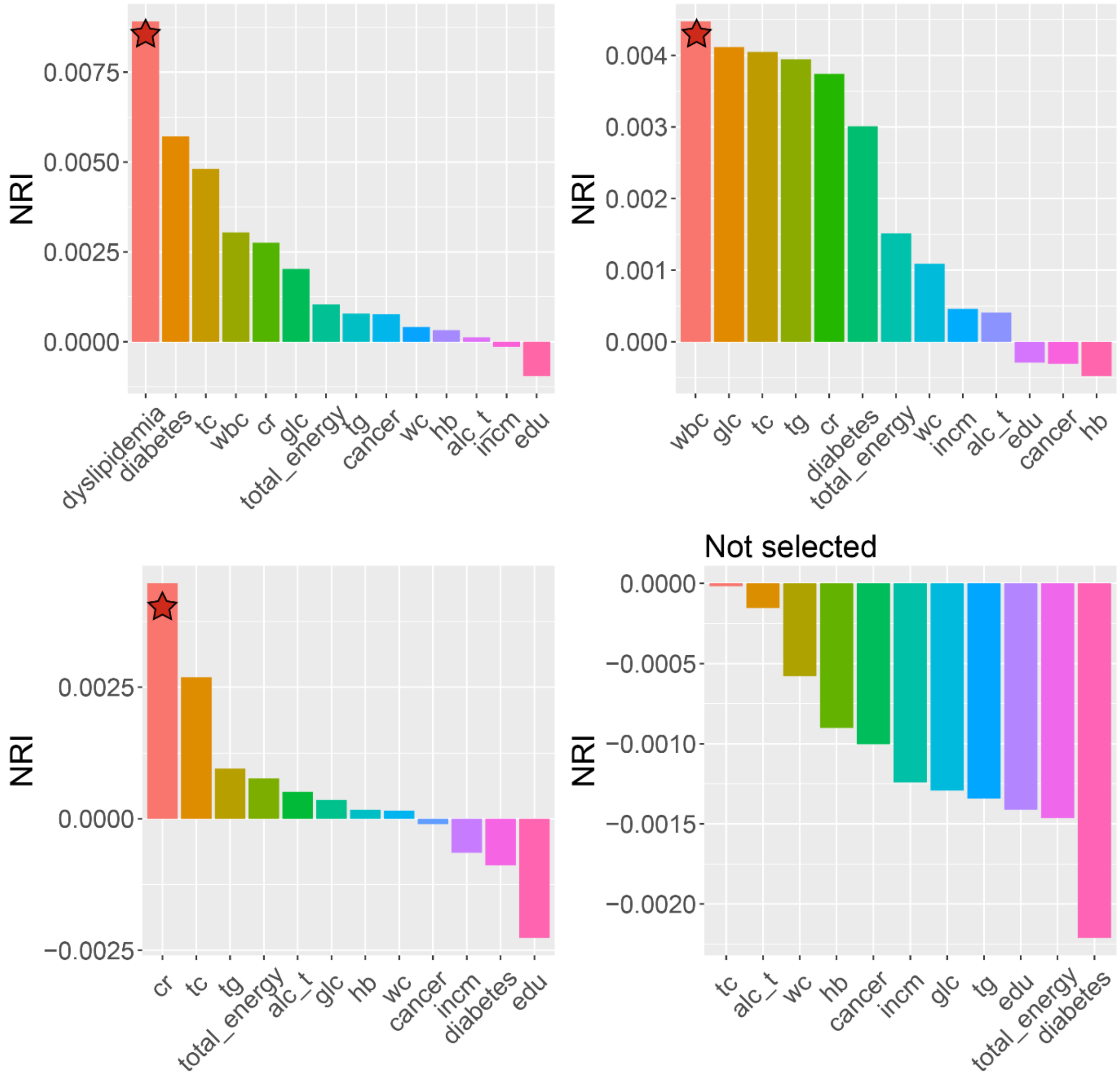

Supplementary Figure S3. CART analysis flowchart

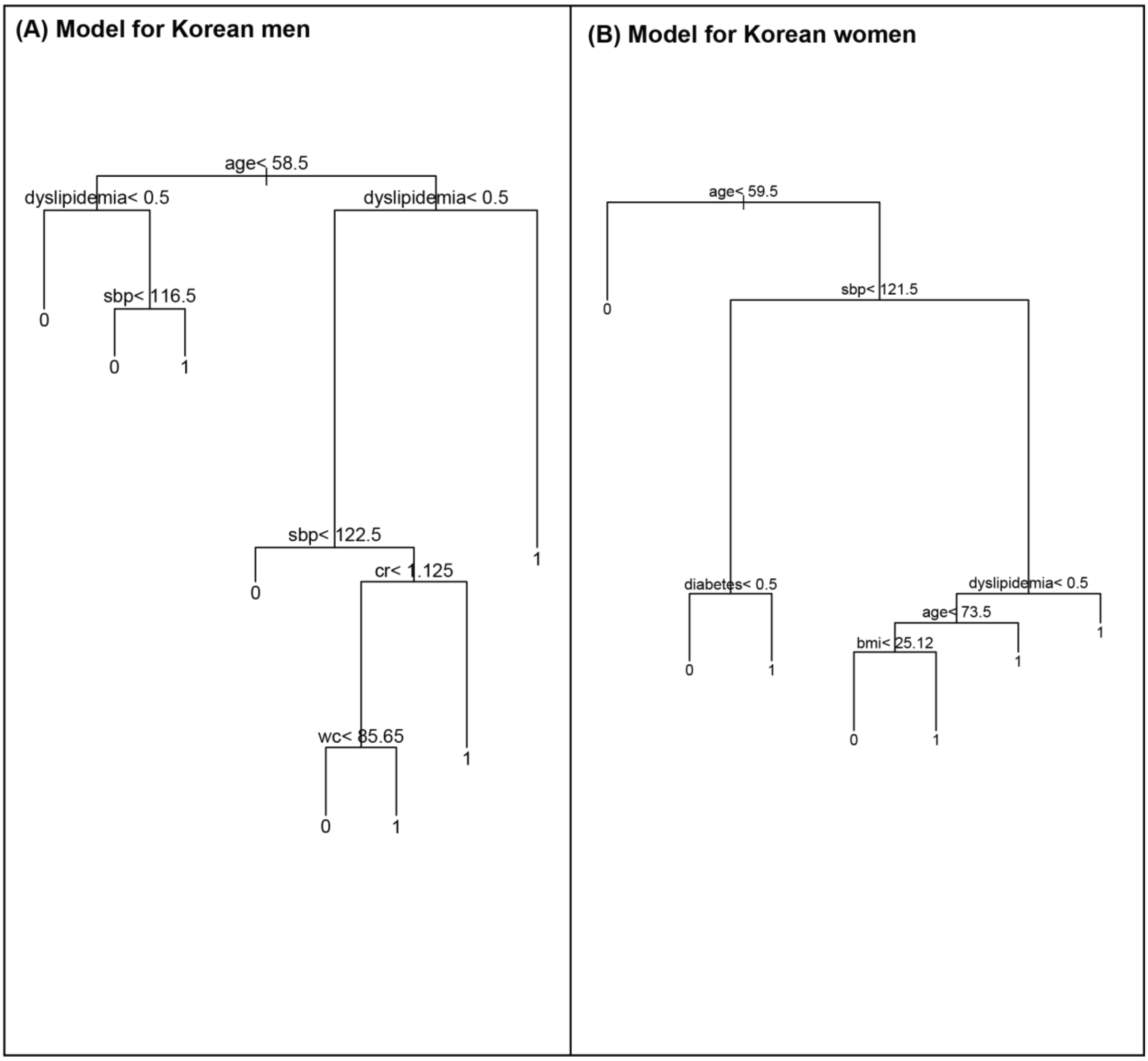

Supplementary Figure S4. Variance importance of the sex-specific hypertension classification model measured by CART method. (A) Men (B) Women.

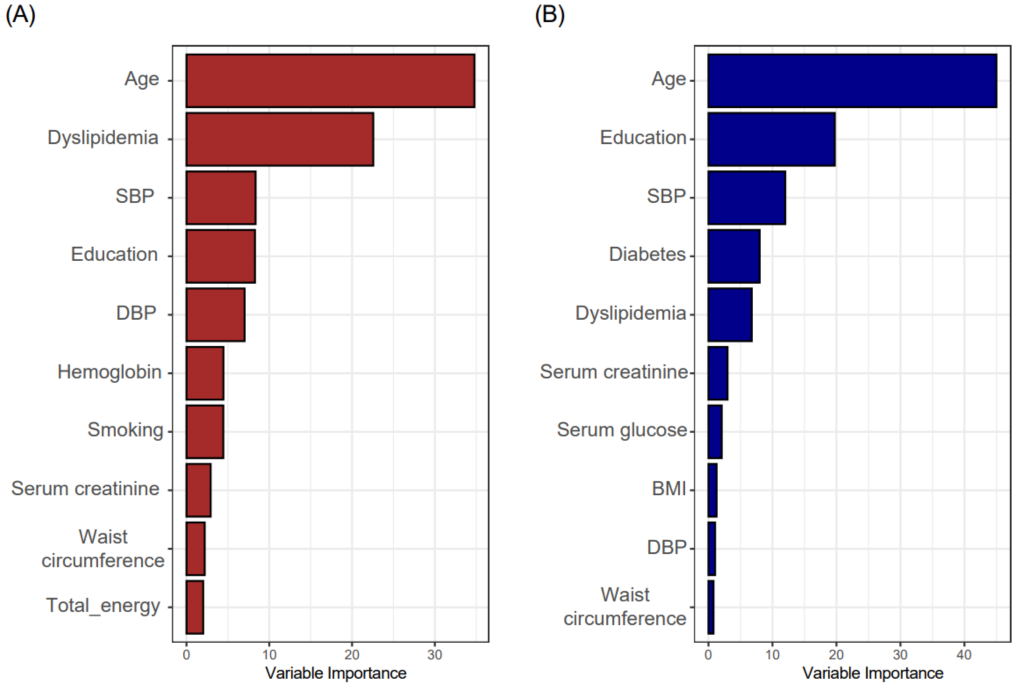

References

1. Buford, T.W. Hypertension and aging. *Ageing Res. Rev.* **2016**, *26*, 96-111, doi:10.1016/j.arr.2016.01.007.
2. Di Giosia, P.; Giorgini, P.; Stamerra, C.A.; Petrarca, M.; Ferri, C.; Sahebkar, A. Gender differences in epidemiology, pathophysiology, and treatment of hypertension. *Curr. Atheroscler. Rep.* **2018**, *20*, 13, doi:10.1007/s11883-018-0716-z.
3. Gao, Y.; Chen, G.; Tian, H.; Lin, L.; Lu, J.; Weng, J.; Jia, W.; Ji, L.; Xiao, J.; Zhou, Z.; et al. Prevalence of hypertension in china: a cross-sectional study. *PLoS One* **2013**, *8*, e65938, doi:10.1371/journal.pone.0065938.
4. Hajjar, I.; Kotchen, T.A. Trends in prevalence, awareness, treatment, and control of hypertension in the United States, 1988-2000. *JAMA* **2003**, *290*, 199-206, doi:10.1001/jama.290.2.199.
5. Pandit, A.U.; Tang, J.W.; Bailey, S.C.; Davis, T.C.; Bocchini, M.V.; Persell, S.D.; Federman, A.D.; Wolf, M.S. Education, literacy, and health: Mediating effects on hypertension knowledge and control. *Patient Educ. Couns.* **2009**, *75*, 381-385, doi:10.1016/j.pec.2009.04.006.
6. Leng, B.; Jin, Y.; Li, G.; Chen, L.; Jin, N. Socioeconomic status and hypertension: a meta-analysis. *J. Hypertens.* **2015**, *33*, 221-229, doi:10.1097/hjh.0000000000000428.
7. Tsimihodimos, V.; Gonzalez-Villalpando, C.; Meigs, J.B.; Ferrannini, E. Hypertension and Diabetes Mellitus: Coprediction and Time Trajectories. *Hypertension* **2018**, *71*, 422-428, doi:10.1161/hypertensionaha.117.10546.
8. Otsuka, T.; Takada, H.; Nishiyama, Y.; Kodani, E.; Saiki, Y.; Kato, K.; Kawada, T. Dyslipidemia and the risk of developing hypertension in a working-age male population. *J. Am. Heart Assoc.* **2016**, *5*, e003053, doi:10.1161/jaha.115.003053.
9. Han, H.; Guo, W.; Shi, W.; Yu, Y.; Zhang, Y.; Ye, X.; He, J. Hypertension and breast cancer risk: a systematic review and meta-analysis. *Sci. Rep.* **2017**, *7*, 44877, doi:10.1038/srep44877.
10. Liang, Z.; Xie, B.; Li, J.; Wang, X.; Wang, S.; Meng, S.; Ji, A.; Zhu, Y.; Xu, X.; Zheng, X.; et al. Hypertension and risk of prostate cancer: a systematic review and meta-analysis. *Sci. Rep.* **2016**, *6*, 31358, doi:10.1038/srep31358.
11. Hidayat, K.; Du, X.; Zou, S.Y.; Shi, B.M. Blood pressure and kidney cancer risk: meta-analysis of prospective studies. *J. Hypertens.* **2017**, *35*, 1333-1344, doi:10.1097/hjh.0000000000001286.
12. Gabin, J.M.; Tambs, K.; Saltvedt, I.; Sund, E.; Holmen, J. Association between blood pressure and Alzheimer disease measured up to 27 years prior to diagnosis: the HUNT Study. *Alzheimers Res. Ther.* **2017**, *9*, 37, doi:10.1186/s13195-017-0262-x.

13. Qiu, C.; Hu, G.; Kivipelto, M.; Laatikainen, T.; Antikainen, R.; Fratiglioni, L.; Jousilahti, P.; Tuomilehto, J. Association of blood pressure and hypertension with the risk of Parkinson disease: the National FINRISK Study. *Hypertension* **2011**, *57*, 1094-1100, doi:10.1161/hypertensionaha.111.171249.

14. Wang, Y.; Zeng, Y.; Lin, C.; Chen, Z. Hypertension and non-alcoholic fatty liver disease proven by transient elastography. *Hepatol. Res.* **2016**, *46*, 1304-1310, doi:10.1111/hepr.12688.

15. Primatesta, P.; Falaschetti, E.; Gupta, S.; Marmot, M.G.; Poulter, N.R. Association between smoking and blood pressure: evidence from the health survey for England. *Hypertension* **2001**, *37*, 187-193, doi:10.1161/01.hyp.37.2.187.

16. Xin, X.; He, J.; Frontini, M.G.; Ogden, L.G.; Motsamai, O.I.; Whelton, P.K. Effects of alcohol reduction on blood pressure: a meta-analysis of randomized controlled trials. *Hypertension* **2001**, *38*, 1112-1117, doi:10.1161/hy1101.093424.

17. Nakanishi, N.; Yoshida, H.; Nakamura, K.; Suzuki, K.; Tatara, K. Alcohol consumption and risk for hypertension in middle-aged Japanese men. *J. Hypertens.* **2001**, *19*, 851-855, doi:10.1097/00004872-200105000-00003.

18. Kelley, G.A.; Kelley, K.S. Progressive resistance exercise and resting blood pressure : A meta-analysis of randomized controlled trials. *Hypertension* **2000**, *35*, 838-843, doi:10.1161/01.hyp.35.3.838.

19. Whelton, S.P.; Chin, A.; Xin, X.; He, J. Effect of aerobic exercise on blood pressure: a meta-analysis of randomized, controlled trials. *Ann. Intern. Med.* **2002**, *136*, 493-503, doi:10.7326/0003-4819-136-7-200204020-00006.

20. Sacks, F.M.; Svetkey, L.P.; Vollmer, W.M.; Appel, L.J.; Bray, G.A.; Harsha, D.; Obarzanek, E.; Conlin, P.R.; Miller, E.R., 3rd; Simons-Morton, D.G.; et al. Effects on blood pressure of reduced dietary sodium and the Dietary Approaches to Stop Hypertension (DASH) diet. DASH-Sodium Collaborative Research Group. *N. Engl. J. Med.* **2001**, *344*, 3-10, doi:10.1056/nejm200101043440101.

21. Vollmer, W.M.; Sacks, F.M.; Ard, J.; Appel, L.J.; Bray, G.A.; Simons-Morton, D.G.; Conlin, P.R.; Svetkey, L.P.; Erlinger, T.P.; Moore, T.J.; et al. Effects of diet and sodium intake on blood pressure: subgroup analysis of the DASH-sodium trial. *Ann. Intern. Med.* **2001**, *135*, 1019-1028, doi:10.7326/0003-4819-135-12-200112180-00005.

22. Appel, L.J.; Brands, M.W.; Daniels, S.R.; Karanja, N.; Elmer, P.J.; Sacks, F.M. Dietary approaches to prevent and treat hypertension: a scientific statement from the American Heart Association. *Hypertension* **2006**, *47*, 296-308, doi:10.1161/01.Hyp.0000202568.01167.B6.

23. Wang, Q.; Xi, B.; Liu, M.; Zhang, Y.; Fu, M. Short sleep duration is associated with hypertension risk among adults: a systematic review and meta-analysis. *Hypertens. Res.* **2012**, *35*, 1012-1018, doi:10.1038/hr.2012.91.

24. Staessen, J.; Bulpitt, C.J.; Fagard, R.; Lijnen, P.; Amery, A. The influence of menopause on blood pressure. *J. Hum. Hypertens.* **1989**, *3*, 427-433.

25. Zanchetti, A.; Facchetti, R.; Cesana, G.C.; Modena, M.G.; Pirrelli, A.; Sega, R. Menopause-related blood pressure increase and its relationship to age and body mass index: the SIMONA epidemiological study. *J. Hypertens.* **2005**, *23*, 2269-2276, doi:10.1097/01.hjh.0000194118.35098.43.

26. Chasan-Taber, L.; Willett, W.C.; Manson, J.E.; Spiegelman, D.; Hunter, D.J.; Curhan, G.; Colditz, G.A.; Stampfer, M.J. Prospective study of oral contraceptives and hypertension among women in the United States. *Circulation* **1996**, *94*, 483-489, doi:10.1161/01.cir.94.3.483.

27. He, J.; Whelton, P.K.; Appel, L.J.; Charleston, J.; Klag, M.J. Long-term effects of weight loss and dietary sodium reduction on incidence of hypertension. *Hypertension* **2000**, *35*, 544-549, doi:10.1161/01.hyp.35.2.544.

28. Neter, J.E.; Stam, B.E.; Kok, F.J.; Grobbee, D.E.; Geleijnse, J.M. Influence of weight reduction on blood pressure: a meta-analysis of randomized controlled trials. *Hypertension* **2003**, *42*, 878-884, doi:10.1161/01.Hyp.0000094221.86888.Ae.

29. Siani, A.; Cappuccio, F.P.; Barba, G.; Trevisan, M.; Farinaro, E.; Lacone, R.; Russo, O.; Russo, P.; Mancini, M.; Strazzullo, P. The relationship of waist circumference to blood pressure: the Olivetti Heart Study. *Am. J. Hypertens.* **2002**, *15*, 780-786, doi:10.1016/s0895-7061(02)02976-x.

30. Vasan, R.S.; Larson, M.G.; Leip, E.P.; Kannel, W.B.; Levy, D. Assessment of frequency of progression to hypertension in non-hypertensive participants in the Framingham Heart Study: a cohort study. *Lancet* **2001**, *358*, 1682-1686, doi:10.1016/s0140-6736(01)06710-1.

31. Leitschuh, M.; Cupples, L.A.; Kannel, W.; Gagnon, D.; Chobanian, A. High-normal blood pressure progression to hypertension in the Framingham Heart Study. *Hypertension* **1991**, *17*, 22-27, doi:10.1161/01.hyp.17.1.22.

32. Shankar, A.; Klein, B.E.; Klein, R. Relationship between white blood cell count and incident hypertension. *Am. J. Hypertens.* **2004**, *17*, 233-239, doi:10.1016/j.amjhyper.2003.11.005.
33. Emamian, M.; Hasanian, S.M.; Tayefi, M.; Bijari, M.; Movahedian Far, F.; Shafiee, M.; Avan, A.; Heidari-Bakavoli, A.; Moohebati, M.; Ebrahimi, M.; et al. Association of hematocrit with blood pressure and hypertension. *J. Clin. Lab. Anal.* **2017**, *31*, e22124, doi:10.1002/jcla.22124.
34. Geva, M.; Shlomai, G.; Berkovich, A.; Maor, E.; Leibowitz, A.; Tenenbaum, A.; Grossman, E. The association between fasting plasma glucose and glycated hemoglobin in the prediabetes range and future development of hypertension. *Cardiovasc. Diabetol.* **2019**, *18*, 53, doi:10.1186/s12933-019-0859-4.
35. Sesso, H.D.; Buring, J.E.; Chown, M.J.; Ridker, P.M.; Gaziano, J.M. A prospective study of plasma lipid levels and hypertension in women. *Arch. Intern. Med.* **2005**, *165*, 2420-2427, doi:10.1001/archinte.165.20.2420.
36. Tohidi, M.; Hatami, M.; Hadaegh, F.; Azizi, F. Triglycerides and triglycerides to high-density lipoprotein cholesterol ratio are strong predictors of incident hypertension in Middle Eastern women. *J. Hum. Hypertens.* **2012**, *26*, 525-532, doi:10.1038/jhh.2011.70.
37. Perneger, T.V.; Nieto, F.J.; Whelton, P.K.; Klag, M.J.; Comstock, G.W.; Szklo, M. A prospective study of blood pressure and serum creatinine. Results from the 'Clue' Study and the ARIC Study. *JAMA* **1993**, *269*, 488-493.
38. Lee, D.H.; Jacobs, D.R., Jr.; Gross, M.; Kiefe, C.I.; Roseman, J.; Lewis, C.E.; Steffes, M. Gamma-glutamyltransferase is a predictor of incident diabetes and hypertension: the Coronary Artery Risk Development in Young Adults (CARDIA) Study. *Clin. Chem.* **2003**, *49*, 1358-1366, doi:10.1373/49.8.1358.
39. Sesso, H.D.; Buring, J.E.; Rifai, N.; Blake, G.J.; Gaziano, J.M.; Ridker, P.M. C-reactive protein and the risk of developing hypertension. *JAMA* **2003**, *290*, 2945-2951, doi:10.1001/jama.290.22.2945.
40. Wang, T.J.; Gona, P.; Larson, M.G.; Levy, D.; Benjamin, E.J.; Tofler, G.H.; Jacques, P.F.; Meigs, J.B.; Rifai, N.; Selhub, J.; et al. Multiple biomarkers and the risk of incident hypertension. *Hypertension* **2007**, *49*, 432-438, doi:10.1161/01.HYP.0000256956.61872.aa.
41. Chamarthi, B.; Williams, G.H.; Ricchiuti, V.; Srikumar, N.; Hopkins, P.N.; Luther, J.M.; Jeunemaitre, X.; Thomas, A. Inflammation and hypertension: the interplay of interleukin-6, dietary sodium, and the renin-angiotensin system in humans. *Am. J. Hypertens.* **2011**, *24*, 1143-1148, doi:10.1038/ajh.2011.113.

42. Vasan, R.S.; Evans, J.C.; Larson, M.G.; Wilson, P.W.; Meigs, J.B.; Rifai, N.; Benjamin, E.J.; Levy, D. Serum aldosterone and the incidence of hypertension in nonhypertensive persons. *N. Engl. J. Med.* **2004**, *351*, 33-41, doi:10.1056/NEJMoa033263.

43. Wang, N.; Shao, H.; Chen, Y.; Xia, F.; Chi, C.; Li, Q.; Han, B.; Teng, Y.; Lu, Y. Follicle-stimulating hormone, its association with cardiometabolic risk factors, and 10-year risk of cardiovascular disease in postmenopausal women. *J. Am. Heart Assoc.* **2017**, *6*, e005918, doi:10.1161/jaha.117.005918.

44. Masi, C.M.; Hawkley, L.C.; Xu, X.; Veenstra, T.D.; Cacioppo, J.T. Serum estrogen metabolites and systolic blood pressure among middle-aged and older women and men. *Am. J. Hypertens.* **2009**, *22*, 1148-1153, doi:10.1038/ajh.2009.155.

45. Fogari, R.; Preti, P.; Zoppi, A.; Fogari, E.; Rinaldi, A.; Corradi, L.; Mugellini, A. Serum testosterone levels and arterial blood pressure in the elderly. *Hypertens. Res.* **2005**, *28*, 625-630, doi:10.1291/hypres.28.625.

46. Jacobs, D.R., Jr.; Yatsuya, H.; Hearst, M.O.; Thyagarajan, B.; Kalhan, R.; Rosenberg, S.; Smith, L.J.; Barr, R.G.; Duprez, D.A. Rate of decline of forced vital capacity predicts future arterial hypertension: the Coronary Artery Risk Development in Young Adults Study. *Hypertension* **2012**, *59*, 219-225, doi:10.1161/hypertensionaha.111.184101.

47. Engström, G.; Wollmer, P.; Valind, S.; Hedblad, B.; Janzon, L. Blood pressure increase between 55 and 68 years of age is inversely related to lung function: longitudinal results from the cohort study 'Men born in 1914'. *J. Hypertens.* **2001**, *19*, 1203-1208, doi:10.1097/00004872-200107000-00004.
